# How much has the prevalence of anemia in Peruvian women changed with the WHO 2024 criteria? analysis of ENDES 2023

**DOI:** 10.17843/rpmesp.2024.413.13993

**Published:** 2024-07-22

**Authors:** Akram Hernández-Vásquez, Jamee Guerra Valencia, Rodrigo Vargas-Fernández

**Affiliations:** 1 San Ignacio de Loyola University, Vice-rectorate for Research, Center of Excellence in Economic and Social Research in Health, Lima, Peru San Ignacio de Loyola University San Ignacio de Loyola University Vice-rectorate for Research Center of Excellence in Economic and Social Research in Health Lima Peru; 2 Facultad de Ciencias de la Salud, Universidad Privada del Norte, Lima, Peru Universidad Privada del Norte Facultad de Ciencias de la Salud Universidad Privada del Norte Lima Peru; 3 Epidemiology and Health Economics Research (EHER), Universidad Científica del Sur, Lima, Peru Universidad Científica del Sur Epidemiology and Health Economics Research (EHER) Universidad Científica del Sur Lima Peru

To the Editor. Anemia is a treatable and preventable condition that significantly affects women of childbearing age, pregnant women, and infants. Worldwide, the prevalence of anemia has slightly decreased in women aged 15 to 49 years (31% in 2010 to 30% in 2019), as well as in pregnant women (41% to 36% in the same period); low- and middle-income countries still maintain the highest prevalence rates ^1^. These figures have led the World Health Organization (WHO) to consider anemia as a problem that needs to be controlled soon, therefore one of its established objectives is a 50% reduction in anemia in women of childbearing age by 2025 ^2^.

Although anemia figures vary according to region, altitude, sex, age and other clinical characteristics, WHO published in 2024 a new guideline for the assessment of anemia and its severity ^3^. One of the novelties of this guideline is the modification of the hemoglobin adjustment for altitude of residence to assess the effect of living in regions below 2500 meters above mean sea level (MASL) ^3^. In comparison with the 1989 US Centers for Disease Control and Prevention (CDC) recommendation to adjust hemoglobin from 1000 MASL ^4^, the new WHO guideline proposes an adjustment of approximately 4 g/L for every 500 MASL ^3^. In addition, a differentiated cut-off point for the second trimester of gestation was included in the new guideline ^3^. Finally, on April 8, 2024, the Peruvian Ministry of Health approved NTS 213-MINSA/DGIESP-2024, which implements the new guideline in Peru ^5^. These changes could modify the anemia figures and the prioritization of public health measures. Therefore, this study aimed to determine changes in the prevalence of anemia in Peruvian women according to the two described criteria.

For this study we used the Demographic and Family Health Survey (ENDES) 2023 and included 31,639 women aged 15-49 years. The analysis was carried out using Stata 18 (StataCorp, College Station, Texas, USA), incorporating strata, clusters and sampling weights. Descriptive measures were used to report anemia prevalences along with their 95% confidence intervals and percentage point (pp) changes. The variable of interest was the presence of anemia. Two anemia conditions were estimated based on the altitude-adjusted hemoglobin concentration of the cluster of residence, using the cutoff points established by the CDC criteria ^4^ and the new WHO criteria ^3^. The adjustments were made according to the altitude of the cluster, applying the CDC criteria from 1000 MASL and the WHO criteria from 500 MASL, with the following equations:

WHO (g/L) =0.0056384 * A+ 0.0000003 * A^2^

CDC (g/L)=-0.32 * (A * 0.0033)+ 0.22 * (A * 0.003)^2^

Where: A=altitude in MASL

By applying the new WHO criteria, we found that the prevalence of anemia in women increased by 0.3 pp. Among pregnant women, the prevalence decreased by 4.2 pp. The prevalence of anemia increased at altitudes between 1000 and 3000 MASL by 9.0 and 5.9 pp, respectively. In contrast, a reduction of up to 18.9 pp was found at altitudes ≥4000 MASL. We found an increase of 3.8 pp in the jungle region. On the other hand, when analyzing by department, the reduction in prevalence in Puno was noteworthy, as it was no longer the department with the highest prevalence of anemia, with a decrease of 12.5 pp (31.8% with CDC vs. 19.3% with WHO). Other changes in different departments are shown in Table 1.

**Table 1:** Prevalence of anemia in Peruvian women according to CDC 1989 and WHO 2024 criteria.

Characteristic		CDC 1989	WHO 2024	Difference*
		% (95% CI)	% (95% CI)	WHO - CDC
Total		22.7 (21.7-23.7)	23.0 (22.0-24.0)	0.3
Anemia level				
	Severe	0.2 (0.2-0.3)	0.7 (0.6-0.9)	0.5
	Moderate	3.4 (3.0-3.9)	7.5 (6.9-8.2)	4.1
	Mild	19.1 (18.1-20.0)	14.8 (14.0-15.5)	-4.3
Age in years				
	15-19	25.0 (22.8-27.4)	25.0 (22.8-27.4)	0.0
	20-49	22.3 (21.2-23.4)	22.6 (21.6-23.7)	0.3
Current pregnancy				
	No	22.7 (21.7-23.7)	23.1 (22.1-24.1)	0.4
	Yes	24.1 (19.5-29.5)	19.9 (15.5-25.1)	-4.2
Welfare quintile				0.0
	Poorest	22.0 (20.5-23.5)	22.5 (21.1-24.0)	0.5
	Poor	20.8 (19.2-22.5)	21.1 (19.5-22.8)	0.3
	Mean	22.6 (20.7-24.6)	22.4 (20.5-24.3)	-0.2
	Rich	24.3 (22.0-26.7)	24.9 (22.6-27.4)	0.6
	Richest	24.0 (21.0-27.2)	24.3 (21.3-27.5)	0.3
Residence altitude (MASL)				
	<500	24.0 (22.6-25.3)	23.8 (22.5-25.2)	-0.2
	≥500-<1000	16.3 (13.0-20.3)	24.4 (20.6-28.7)	8.1
	≥1000-<2000	15.5 (13.1-18.2)	24.5 (21.6-27.6)	9.0
	≥2000-<3000	15.8 (14.0-17.9)	21.7 (19.6-24.0)	5.9
	≥3000-<4000	25.8 (23.7-28.1)	19.6 (17.6-21.6)	-6.2
	≥4000	33.4 (27.9-39.4)	14.5 (11.1-18.7)	-18.9
Residence area				
	Urban	22.9 (21.7-24.1)	23.1 (22.0-24.3)	0.2
	Rural	22.0 (20.6-23.5)	22.5 (21.1-24.0)	0.5
Natural region				
	Coast	23.5 (22.0-25.0)	23.9 (22.4-25.4)	0.4
	Highlands	21.1 (19.7-22.5)	19.2 (17.9-20.5)	-1.9
	Jungle	22.1 (20.7-23.7)	25.9 (24.5-27.4)	3.8
Department				
	Amazonas	16.8 (13.5-20.6)	21.0 (17.6-24.9)	4.2
	Ancash	19.4 (15.9-23.5)	19.6 (16.1-23.6)	0.2
	Apurímac	17.7 (14.0-22.1)	17.6 (12.7-23.8)	-0.1
	Arequipa	18.1 (15.1-21.6)	22.7 (19.3-26.5)	4.6
	Ayacucho	20.1 (16.4-24.5)	20.7 (17.0-25.0)	0.6
	Cajamarca	11.4 (9.2-14.1)	17.0 (14.4-19.9)	5.6
	Callao	22.5 (19.1-26.5)	22.3 (18.8-26.2)	-0.2
	Cusco	26.9 (22.2-32.1)	21.0 (16.9-25.7)	-5.9
	Huancavelica	20.1 (17.0-23.6)	15.0 (12.1-18.3)	-5.1
	Huánuco	14.8 (12.1-18.1)	20.1 (17.1-23.6)	5.3
	Ica	17.9 (15.2-21.0)	18.0 (15.3-20.9)	0.1
	Junín	25.4 (21.7-29.5)	25.5 (21.9-29.5)	0.1
	La Libertad	22.5 (19.3-26.0)	22.8 (19.6-26.3)	0.3
	Lambayeque	23.4 (20.4-26.7)	23.7 (20.5-27.1)	0.3
	Lima	24.4 (22.1-26.8)	24.6 (22.3-27.1)	0.2
	Loreto	29.4 (26.2-32.7)	29.2 (26.0-32.5)	-0.2
	Madre de Dios	27.4 (24.5-30.5)	27.3 (24.4-30.5)	-0.1
	Moquegua	20.9 (17.0-25.4)	27.5 (23.0-32.5)	6.6
	Pasco	25.0 (21.3-29.1)	20.5 (16.9-24.6)	-4.5
	Piura	23.3 (19.8-27.3)	24.1 (20.6-28.1)	0.8
	Puno	31.8 (27.0-37.0)	19.3 (15.5-23.8)	-12.5
	San Martin	15.9 (13.2-19.0)	19.9 (17.1-23.0)	4.0
	Tacna	20.3 (16.8-24.3)	26.1 (22.3-30.2)	5.8
	Tumbes	26.0 (22.5-29.8)	25.8 (22.4-29.6)	-0.2
	Ucayali	27.6 (24.3-31.3)	27.8 (24.3-31.6)	0.2

CDC: US Centers for Disease Control and Prevention, WHO: World Health Organization, CI: confidence interval.

All estimates included the characteristics of the complex sample design.

Callao refers to the Constitutional Province of Callao.

The welfare quintile is calculated by the National Institute of Statistics and Informatics from a welfare index. This index is calculated using data on household possessions, such as televisions, materials used for housing construction, type of access to water and sanitation facilities.

* The difference is reported in percentage points from the subtraction of the prevalence with the WHO 2024 criterion minus CDC 1989.

All estimates obtained a coefficient of variation of less than 15%.

The hemoglobinometer HemoCue® model Hb 201+ was used for hemoglobin analysis in the Demographic and Family Health Survey.

Our results show that the implementation of the new WHO guidelines will lead to a change in the prevalence of anemia in pregnant women and women living at different altitudes. It is noteworthy that the prevalence of anemia increased at altitudes below 3000 MASL, while it decreased from 3000 MASL onwards. This finding is reaffirmed by the fact that Puno, one of the departments with the highest altitude, went from being the department with the highest prevalence to presenting figures that are even lower than those of Lima, leaving Loreto as the department with the highest prevalence (29.2% with WHO criteria). These findings are explained by the difference between CDC and WHO for hemoglobin adjustments. While at altitudes >3194.2 MASL the WHO 2024 guidelines make a lower adjustment compared to CDC, at altitudes <3194.2 MASL WHO recommends a higher adjustment ^3^ (see Figure in Supplementary Material). In addition, the latter is more pronounced between 1000 and 2500 MASL. This explains the increase in prevalence in departments with populations residing in this altitude range, such as San Martín and Amazonas. All of the above suggests that the effect of altitude may have been overestimated to date, which could have implications for the allocation of resources to address the public health burden generated by anemia.

In conclusion, the implementation of the new WHO criteria significantly changes the prevalence of anemia in Peruvian women, especially at altitudes above 3000 MASL and in departments at high altitude. This indicates that previous estimates may have overestimated hemoglobin levels in Peruvian women. Finally, our results underscore the need to accurately determine normative hemoglobin values in our population using markers such as serum ferritin. Likewise, it will be necessary to rethink public health strategies and the allocation of resources to more accurately address the burden of anemia in the country and “leave no one behind”.
