# Supplementary material for: How much has the prevalence of anemia in Peruvian women changed with the WHO 2024 criteria? analysis of ENDES 2023
Source: Rev Peru Med Exp Salud Publica. 2024 Jul 22;41(3):324–6. doi: 10.17843/rpmesp.2024.413.13993 (PMC11495922; doi:10.17843/rpmesp.2024.413.13993)
Supplement: Supplementary material. — Available in the electronic version of the RPMESP. [file rpmesp-41-03-13993-s001.docx]

**MATERIAL SUPLEMENTARIO**

**
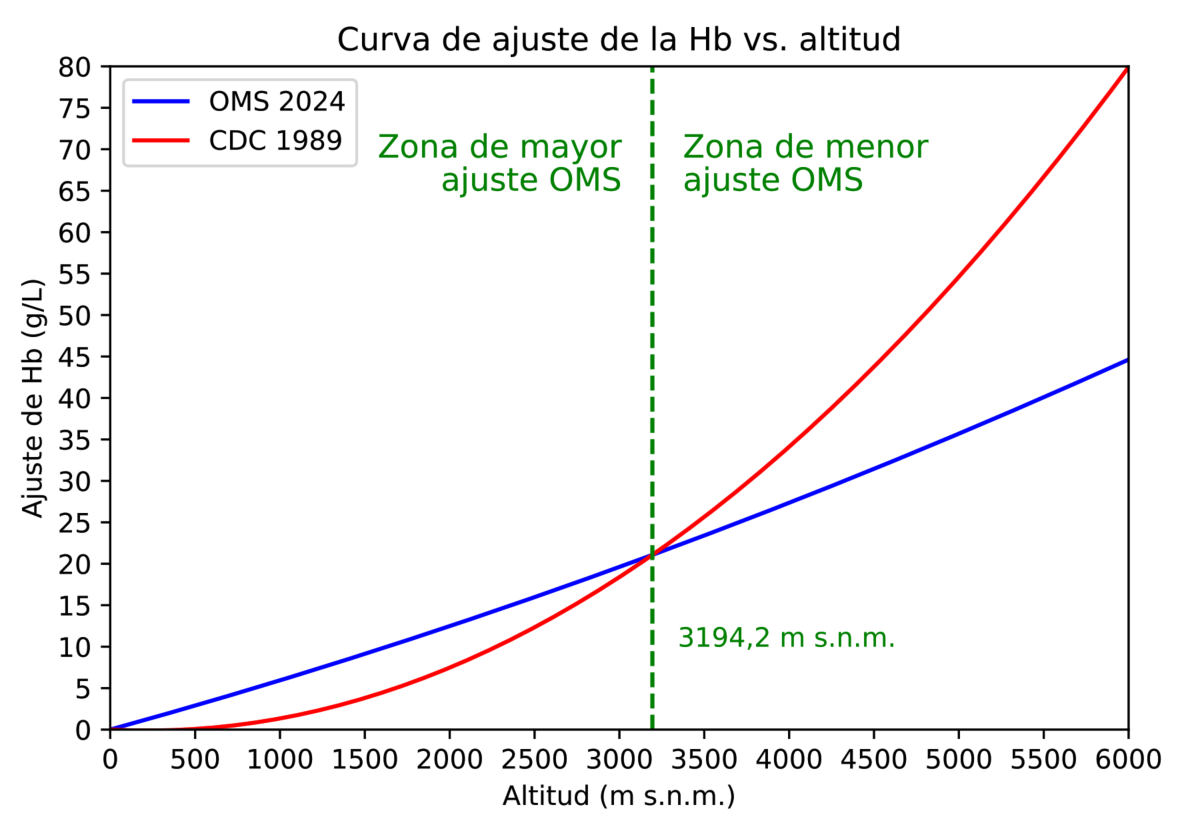
**

**Figura 1:** Curvas de ajuste de la hemoglobina vs. altitud según las ecuaciones de regresión de CDC 1989 y OMS 2024.

***Fuente*:** Elaboración propia a partir de las ecuaciones de regresión de CDC 1989 ^(1)^ y OMS 2024 ^(2)^.

En la siguiente página se describe el código en Python utilizado para generar esta figura en *Visual Studio Code*. La línea vertical representa el valor de intersección de las curvas de ajustes para CDC 1989 y OMS 2024 (3194,2 m s. n. m.)

CDC: *Centers for Disease Control and Prevention*; OMS: Organización Mundial de la Salud; m s. n. m.: metros sobre el nivel del mar; Hb: Hemoglobina.

**REFERENCIAS BIBLIOGRÁFICAS**

1. Centers for Disease Control (CDC). CDC criteria for anemia in children and childbearing-aged women. MMWR Morb Mortal Wkly Rep. 1989;38(22):400–4.
2. World Health Organization. Guideline on haemoglobin cutoffs to define anaemia in individuals and populations. 2024; Disponible en: https://iris.who.int/bitstream/handle/10665/376196/9789240088542-eng.pdf?sequence=1

| **Código en Python para Visual Studio Code** | |
| --- | --- |
| 1  2  3  4  5  6  7  8  9  10  11  12  13  14  15  16  17  18  19  20  21  22  23  24  25  26  27  28  29  30  31  32  33  34  35  36  37  38  39  40  41 | import numpy as np  import matplotlib.pyplot as plt  # Generar valores de A y altitud de 0 a 5000 en incrementos de 500  A = np.arange(0, 6001)  altitud = A  # Calcular los valores de OMS  OMS = 0.0056384 * A + 0.0000003 * A**2  # Calcular los valores de CDC  factor = altitud * 0.0033  CDC = -0.32 * factor + 0.22 * factor**2  # Crear el gráfico  plt.figure(figsize=(10, 6))  plt.plot(A, OMS, color='blue', label='OMS 2024')  plt.plot(A, CDC, color='red', label='CDC 1989')  # Personalizar el gráfico  plt.title("Curva de ajuste de la Hb vs. altitud")  plt.ylabel("Ajuste de Hb (g/L)")  plt.xlabel("Altitud (m s.n.m.)")  plt.ylim(0, 80)  plt.xlim(0, 6000)  plt.axvline(x=3194.2, color='green', linestyle='--')  plt.text(3194.2, 10, " 3194,2 m s.n.m.", horizontalalignment='left', color='green', fontsize=10)  plt.text(3194.2, 69, "Zona de mayor ", horizontalalignment='right', color='green', fontsize=12)  plt.text(3194.2, 65, "ajuste OMS ", horizontalalignment='right', color='green', fontsize=12)  plt.text(3194.2, 69, " Zona de menor", horizontalalignment='left', color='green', fontsize=12)  plt.text(3194.2, 65, " ajuste OMS", horizontalalignment='left', color='green', fontsize=12)  plt.legend()  # Establecer los intervalos en el eje x de 500 en 500  plt.xticks(np.arange(0, 6001, 500))  # Establecer los intervalos en el eje y de 5 en 5  plt.yticks(np.arange(0, 81, 5))  # Mostrar el gráfico  plt.show() |
