# Supplementary material for: How much has the prevalence of anemia in Peruvian women changed with the WHO 2024 criteria? analysis of ENDES 2023
Source: Rev Peru Med Exp Salud Publica. 2024 Jul 22;41(3):324–6. doi: 10.17843/rpmesp.2024.413.13993 (PMC11495922; doi:10.17843/rpmesp.2024.413.13993)
Supplement: Translation

# Translation: ¿Cuánto ha cambiado la prevalencia de anemia en mujeres peruanas con los criterios de la OMS 2024? análisis de la ENDES 2023

**Authors:** Akram Hernández-Vásquez, Jamee Guerra Valencia, Rodrigo Vargas-Fernández

**Affiliations:** 1 Universidad San Ignacio de Loyola, Vicerrectorado de Investigación, Centro de Excelencia en Investigaciones Económicas y Sociales en Salud, Lima, Perú.; 2 Facultad de Ciencias de la Salud, Universidad Privada del Norte, Lima, Perú.; 3 Epidemiology and Health Economics Research (EHER), Universidad Científica del Sur, Lima, Perú.

Sr. Editor. La anemia es una condición tratable y prevenible que afecta significativamente a mujeres en edad fértil, gestantes e infantes. A nivel mundial, la prevalencia de anemia en mujeres de 15 a 49 años ha mostrado una ligera disminución (31% en 2010 a 30% en 2019), al igual que en gestantes (41% a 36% en el mismo período), donde los países de bajos y medianos ingresos aún mantienen las mayores prevalencias ^1^. Estas cifras han llevado a la Organización Mundial de la Salud (OMS) a considerar la anemia como un problema que requiere un control acelerado, estableciendo como uno de sus objetivos la reducción del 50% de la anemia en mujeres en edad fértil al 2025 ^2^.

Si bien las cifras de anemia varían según la región, altitud, sexo, edad y otras características clínicas, la OMS publicó en 2024 una nueva guía para la evaluación de la anemia y su severidad ^3^. Una de las novedades de esta guía es la modificación del ajuste de hemoglobina por altitud de residencia para evaluar el efecto de vivir en regiones menores a 2500 metros sobre el nivel del mar (m s. n. m.) ^3^. En comparación con lo establecido por el *US Centers for Disease Control and Prevention (CDC)* en 1989 que recomendaba ajustar la hemoglobina a partir de los 1000 m s. n. m. ^4^, la nueva guía de la OMS propone un ajuste de aproximadamente 4 g/L por cada 500 m s. n. m. ^3^. Además, se incorporó en la nueva guía un punto de corte diferenciado para el segundo trimestre de gestación ^3^. Finalmente, el 8 de abril de 2024, el Ministerio de Salud aprobó la NTS 213-MINSA/DGIESP-2024 que implementa la nueva guía en el Perú ^5^. Estos cambios podrían modificar las cifras de anemia y la priorización de las medidas de salud pública. Por ello, el objetivo del presente estudio fue determinar los cambios en la prevalencia de anemia en mujeres peruanas según los dos criterios descritos.

Se utilizó la Encuesta Demográfica y de Salud Familiar (ENDES) 2023 y se incluyó a un total de 31639 mujeres de 15 a 49 años en el presente estudio. El análisis se llevó a cabo mediante Stata 18 (StataCorp, College Station, Texas, USA), incorporando estratos, conglomerados y pesos muestrales. Se emplearon medidas descriptivas para informar las prevalencias de anemia junto con sus intervalos de confianza al 95% y los cambios en puntos porcentuales (pp). La variable de interés fue la presencia de anemia. Se estimaron dos condiciones de anemia basadas en la concentración de hemoglobina ajustada por altitud del conglomerado de residencia, utilizando los puntos de corte establecidos por los criterios del CDC ^4^ y los nuevos criterios de la OMS ^3^. Los ajustes se realizaron según la altitud del conglomerado, aplicando los criterios del CDC a partir de 1000 m s. n. m. y de la OMS a partir de 500 m s. n. m., con las siguientes ecuaciones:

OMS (g/L)= 0,0056384 * A + 0,0000003* A^2^

CDC (g/L)=- 0,32 * (A * 0,0033) + 0,22 * (A * 0,003)^2^

Donde: A= altitud en m s. n. m.

Se encontró que con los nuevos criterios de la OMS, la prevalencia de anemia en mujeres tuvo un aumento de 0,3 pp. Entre las gestantes, la prevalencia se redujo en 4,2 pp. La prevalencia de anemia aumentó en altitudes entre los 1000 y 3000 m s. n. m. en 9,0 y 5,9 pp, respectivamente. Contrariamente, se observó una reducción de hasta 18,9 pp en altitudes ≥4000 m s. n. m. En la selva, se observó un aumento de 3,8 pp. Por otro lado, en el análisis por departamentos destacó la reducción de prevalencia en Puno que dejó de ser el departamento con mayor prevalencia de anemia con un descenso de 12,5 pp (31,8% con CDC vs*.* 19,3% con OMS). Otros cambios en diferentes departamentos se observan en la Tabla 1.

**Tabla 1:** Prevalencia de anemia en mujeres peruanas según los criterios CDC 1989 y OMS 2024.

Característica		CDC 1989	OMS 2024	Diferencia*
		% (IC 95%)	% (IC 95%)	OMS - CDC
Total		22,7 (21,7-23,7)	23,0 (22,0-24,0)	0,3
Nivel de anemia				
	Grave	0,2 (0,2-0,3)	0,7 (0,6-0,9)	0,5
	Moderado	3,4 (3,0-3,9)	7,5 (6,9-8,2)	4,1
	Leve	19,1 (18,1-20,0)	14,8 (14,0-15,5)	-4,3
Edad en años				
	15-19	25,0 (22,8-27,4)	25,0 (22,8-27,4)	0,0
	20-49	22,3 (21,2-23,4)	22,6 (21,6-23,7)	0,3
Embarazo actual				
	No	22,7 (21,7-23,7)	23,1 (22,1-24,1)	0,4
	Sí	24,1 (19,5-29,5)	19,9 (15,5-25,1)	-4,2
Quintil de bienestar				0,0
	Más pobre	22,0 (20,5-23,5)	22,5 (21,1-24,0)	0,5
	Pobre	20,8 (19,2-22,5)	21,1 (19,5-22,8)	0,3
	Medio	22,6 (20,7-24,6)	22,4 (20,5-24,3)	-0,2
	Rico	24,3 (22,0-26,7)	24,9 (22,6-27,4)	0,6
	Más rico	24,0 (21,0-27,2)	24,3 (21,3-27,5)	0,3
Altitud de residencia (m s. n. m.)				
	<500	24,0 (22,6-25,3)	23,8 (22,5-25,2)	-0,2
	≥500-<1000	16,3 (13,0-20,3)	24,4 (20,6-28,7)	8,1
	≥1000-<2000	15,5 (13,1-18,2)	24,5 (21,6-27,6)	9,0
	≥2000-<3000	15,8 (14,0-17,9)	21,7 (19,6-24,0)	5,9
	≥3000-<4000	25,8 (23,7-28,1)	19,6 (17,6-21,6)	-6,2
	≥4000	33,4 (27,9-39,4)	14,5 (11,1-18,7)	-18,9
Área de residencia				
	Urbano	22,9 (21,7-24,1)	23,1 (22,0-24,3)	0,2
	Rural	22,0 (20,6-23,5)	22,5 (21,1-24,0)	0,5
Región natural				
	Costa	23,5 (22,0-25,0)	23,9 (22,4-25,4)	0,4
	Sierra	21,1 (19,7-22,5)	19,2 (17,9-20,5)	-1,9
	Selva	22,1 (20,7-23,7)	25,9 (24,5-27,4)	3,8
Departamento				
	Amazonas	16,8 (13,5-20,6)	21,0 (17,6-24,9)	4,2
	Ancash	19,4 (15,9-23,5)	19,6 (16,1-23,6)	0,2
	Apurímac	17,7 (14,0-22,1)	17,6 (12,7-23,8)	-0,1
	Arequipa	18,1 (15,1-21,6)	22,7 (19,3-26,5)	4,6
	Ayacucho	20,1 (16,4-24,5)	20,7 (17,0-25,0)	0,6
	Cajamarca	11,4 (9,2-14,1)	17,0 (14,4-19,9)	5,6
	Callao	22,5 (19,1-26,5)	22,3 (18,8-26,2)	-0,2
	Cusco	26,9 (22,2-32,1)	21,0 (16,9-25,7)	-5,9
	Huancavelica	20,1 (17,0-23,6)	15,0 (12,1-18,3)	-5,1
	Huánuco	14,8 (12,1-18,1)	20,1 (17,1-23,6)	5,3
	Ica	17,9 (15,2-21,0)	18,0 (15,3-20,9)	0,1
	Junín	25,4 (21,7-29,5)	25,5 (21,9-29,5)	0,1
	La Libertad	22,5 (19,3-26,0)	22,8 (19,6-26,3)	0,3
	Lambayeque	23,4 (20,4-26,7)	23,7 (20,5-27,1)	0,3
	Lima	24,4 (22,1-26,8)	24,6 (22,3-27,1)	0,2
	Loreto	29,4 (26,2-32,7)	29,2 (26,0-32,5)	-0,2
	Madre de Dios	27,4 (24,5-30,5)	27,3 (24,4-30,5)	-0,1
	Moquegua	20,9 (17,0-25,4)	27,5 (23,0-32,5)	6,6
	Pasco	25,0 (21,3-29,1)	20,5 (16,9-24,6)	-4,5
	Piura	23,3 (19,8-27,3)	24,1 (20,6-28,1)	0,8
	Puno	31,8 (27,0-37,0)	19,3 (15,5-23,8)	-12,5
	San Martin	15,9 (13,2-19,0)	19,9 (17,1-23,0)	4,0
	Tacna	20,3 (16,8-24,3)	26,1 (22,3-30,2)	5,8
	Tumbes	26,0 (22,5-29,8)	25,8 (22,4-29,6)	-0,2
	Ucayali	27,6 (24,3-31,3)	27,8 (24,3-31,6)	0,2

CDC: US Centers for Disease Control and Prevention, OMS: Organización Mundial de la Salud, IC: intervalo de confianza.

Todas las estimaciones incluyeron las características del diseño muestral complejo.

Callao se refiere a la Provincial Constitucional del Callao.

El quintil de bienestar es calculado por el Instituto Nacional de Estadística e Informática a partir de un índice de bienestar. Este índice se calcula utilizando datos sobre la posesión del hogar, como televisores, materiales utilizados para la construcción de viviendas, tipo de acceso al agua y de instalaciones sanitarias.

* La diferencia se informa en puntos porcentuales a partir de la resta de la prevalencia con el criterio OMS 2024 menos CDC 1989.

Todas las estimaciones obtuvieron un coeficiente de variación menor al 15%.

Para el análisis de hemoglobina en la Encuesta Demográfica y de Salud Familiar se utilizó el hemoglobinómetro HemoCue® modelo Hb 201+.

Los resultados presentados muestran que la implementación de la nueva guía de la OMS supondrá un cambio en la prevalencia de anemia en gestantes y mujeres residentes de diferentes altitudes. Destaca que la prevalencia de anemia aumentó a altitudes menores a 3000 m s. n. m., mientras que a partir de 3000 m s. n. m. se redujo. Este hallazgo se reafirma al observar que Puno, uno de los departamentos con mayor altitud, pasó de ser el departamento con mayor prevalencia a presentar cifras que incluso son inferiores a las de Lima, quedando Loreto como el departamento con la mayor prevalencia (29,2% con OMS). Estos hallazgos se explican por la diferencia entre CDC y OMS para los ajustes de hemoglobina. Mientras que a altitudes >3194,2 m s. n. m. las guías de la OMS 2024 realizan un menor ajuste en comparación con CDC, a altitudes <3194,2 m s. n. m. la OMS recomienda un mayor ajuste ^3^ (ver Figura en Material suplementario). Además, este último se acentúa entre los 1000 a 2500 m s. n. m. Esto explica el incremento de la prevalencia en departamentos con población residente en este rango de altitud, tales como San Martín y Amazonas. Todo lo anterior sugiere que hasta la fecha se podría haber sobreestimado el efecto de la altitud, lo que podría tener implicancias en la asignación de recursos para enfrentar la carga de salud pública que genera la anemia.

En conclusión, la implementación de los nuevos criterios de la OMS genera cambios significativos en la prevalencia de anemia en mujeres peruanas, especialmente en altitudes superiores a 3000 m s. n. m. y en departamentos con elevada altitud. Esto indica que las estimaciones anteriores podrían haber sobrestimado los niveles de hemoglobina en las mujeres peruanas. Finalmente, nuestros resultados subrayan la necesidad de determinar de forma precisa los valores normativos de hemoglobina en nuestra población a partir del uso de marcadores tales como la ferritina sérica. Asimismo, será necesario replantear las estrategias de salud pública y la asignación de recursos para enfrentar de manera más precisa la carga de anemia en el país y «no dejar a nadie atrás».
